# Durability performance of alkali-activated concrete with pre-treated coarse recycled aggregates for pavements

**DOI:** 10.1038/s41598-024-64506-6

**Published:** 2024-06-14

**Authors:** MD Ikramullah Khan, V. Vinayaka Ram, Vipulkumar Ishvarbhai Patel

**Affiliations:** 1 Department of Civil Engineering, Birla Institute of Technology and Science – Pilani, Hyderabad Campus, Secunderabad, Telangana 500078 India; 2 School Computing, Engineering and Mathematical Sciences, La Trobe University, Bendigo, VIC 3552 Australia

**Keywords:** Alkali activated concrete, Sea water, Acid resistance, Sorptivity, Compressive strength, Microstructure analysis, Civil engineering, Structural materials

## Abstract

This study examines the effect of coarse recycled aggregates (CRAs) and processed coarse recycled aggregates (PCRAs) on the behaviour of alkali-activated concrete (AAC) before and after exposure to marine seawater and acidic environments (5% HCl and 5% H_2_SO4 solutions). Measurements of compressive strength and the microstructure changes were conducted over periods of 56 and 90 days to assess these effects. The experimental design included varying the replacement levels of NAs with CRAs and PCRAs from (0–100%) and using ground-granulated blast furnace slag and fly ash as constant components. In addition to durability tests, sorptivity assessments were conducted to gauge the material’s porosity and water absorption capabilities. Advanced microstructure techniques, such as scanning electron microscopy (SEM) and X-ray diffraction (XRD), were employed to detail the pre and post-exposure mineralogical and microstructural transformations within the AAC blends. The AAC mixtures incorporating PCRAs emerged as durable, showcasing better strength and a denser, more compact matrix facilitated by the synergistic formation of NASH and CASH gels after exposure to aggressive agents compared to untreated CRAs. In addition, the results show that the samples exposed to marine seawater exhibited improved mechanical performance compared to those exposed to acidic environments. The novelty of this study lies in its exploration of the effects of recycling plant-based CRAs and PCRAs on AAC for marine and acid exposure.

## Introduction

The growing demand for concrete due to urbanization has increased the production of Ordinary Portland Cement (OPC) by over four billion tons in 2018–2020^1–4^. Natural coarse aggregate, comprising 65% of the volume of concrete, is a significant component. Nevertheless, the combined stockpiles are diminishing rapidly. The global natural aggregate (NA) output is around 4.5 billion tonnes. The greenhouse gas emissions associated with producing natural coarse aggregate are estimated to be around 7.4 to 8.0 kg CO_2_-e per tonne^5,6^. Furthermore, releasing dust and particulate matter from trucks and crushers is an additional factor contributing to the escalation of global warming. Moreover, the emissions of dust and particulate matter from trucks and crushers also contribute to the rise in global warming^7^.

This has resulted in using coarse recycled aggregates (CRAs) from constructing and demolishing waste (CDW) to progress towards environmental sustainability and a society with zero carbon emissions. To reduce the need for OPC as a binder, geological source materials with a high silicon and aluminium content or an industrial by-product such as silica fume, nano silica, ground granulated blast furnace slag (GGBFS) and fly ash (FA) react with an alkali activated solution (AAS) called alkali-activated concrete (AAC). A significant portion of CO_2_ emissions during the cement process comes from the calcination of limestone (calcium carbonate, CaCO_3_). When limestone is heated in a cement kiln to produce lime (calcium oxide, CaO), a key ingredient of cement, CO_2_ is released as a byproduct of this chemical reaction^8^. In contrast, producing commercial through-products emits fewer greenhouse gases than OPC. FA emits 80–90%, while GGBFS emits 80% less greenhouse gases than OPC^9–13^.

The use of a mixture of sodium or potassium-based hydroxide and silicate as an alkaline activator has been utilized in research^14,15^. High calcium-based components have been used to enhance the compressive strength of geopolymer concrete, leading to AAC production. Similar to geopolymer concrete, AAC is produced with the addition of calcium carbonate compounds, resulting in the formation of calcium silicate hydrate (CSH) gel, and other gels such as calcium aluminate silicate hydrate (CASH) and sodium aluminate silicate hydrate (NASH)^16–21^.

The potential of CDW as an alternative to NA suggests that CRA can effectively substitute for coarse and fine aggregates^22,23^. Previous research^24,25^, identified the adhered mortar on CRAs as a significant concern due to a weak interfacial transition zone (ITZ), compromising the binder-CRA bond and affecting the concrete’s strength and durability. Studies have shown that completely replacing NA with CRAs in AAC might decrease compressive strength by up to 30%^26,27^. The additional water utilised in the process of CRAs decreases the speed at which the aluminosilicate precursors of an alkali-activated matrix dissolve^28^. Sata et al.^29^ demonstrated that including CRAs results in AAC’s compromised interfacial transition zone. In addition, the sorptivity, water absorption, and volume of permeable voids of AAC rose as the CRA content increased^30,31^. Hu et al.^31^ found strong relationships between the volume of permeable voids, sorptivity and water absorption in AAC with CRAs. Research has shown that the drying shrinkage of AAC steadily increases over time but at a reduced pace after 28 days of curing^32^. Zhang et al.^33^ demonstrated that the drying shrinkage doubled when 100% of the NA was replaced with CRAs. The chemical durability of AAC was examined by subjecting it to seawater, magnesium sulphate, and sulfuric acid. The study indicated that sulfuric acid posed the most risk to the AAC^34^. To address this, a novel and concise hybrid pre-treatment process for CRAs involving mild chemical treatment followed by mechanical treatment was developed, as documented in a prior study by the current research team^35^.

This study investigates AAC’s durability and microstructural characterization performance developed from Na_2_O dosage of 4% and Ms of 1.25. The influence of varying CRAs and PCRA on the AAC sample’s performance was investigated. Various experiments, such as sorptivity, acid, and seawater resistance, are conducted. In addition, microstructural characterization like X-ray diffraction (XRD), Fourier transform infrared spectroscopy (FTIR), stereomicroscopy, scanning electron microscopy (SEM), and energy-dispersive X-ray (EDX) are also conducted to correlate the corresponding observations with the durability performance of the AAC specimens.

## Research significance

The significance of this research lies in its focused examination of AAC incorporating CRAs sourced from CDW recycling plants, particularly under marine and acid exposure conditions. Previous studies have concentrated mainly on the partial substitution of natural aggregates (NAs) with laboratory-controlled CRAs, often overlooking the heterogeneity of CRAs derived from recycling plants and their effect on the durability of AAC. This gap highlights a critical need for an in-depth analysis of AAC’s performance when integrated with recycling plant-based CRAs and PCRAs, extending beyond the conventional laboratory settings to include rigorous marine and acidic environmental testing. This study also aims to assess the sorptivity of AAC with NA, CRAS and PCRAs. This study sheds light on the interaction within AAC mixes, including CDW plant-based CRAs and PCRs, offering valuable insights into their behaviour and performance in challenging environments. The investigation extends to a detailed comparison of AAC’s properties when mixed with CRAs and PCRAs, aiming to delineate the material’s resilience, structural integrity, and longevity. The outcomes of this research are poised to significantly contribute to the construction industry’s knowledge base, providing a robust framework for understanding the potential of recycling plant-based CRAs and PCRAs in AAC applications. By elucidating AAC’s durability and microstructural attributes in marine and acidic conditions, this work paves the way for more sustainable construction practices, encouraging the adoption of environmentally friendly materials that reduce reliance on natural resources. Furthermore, the findings from this study are expected to increase confidence among industry practitioners and researchers alike, fostering the integration of sustainable aggregates into construction processes and promoting the broader application of AAC in infrastructure development.

## Experimental program

### Materials

**Figure 1:**
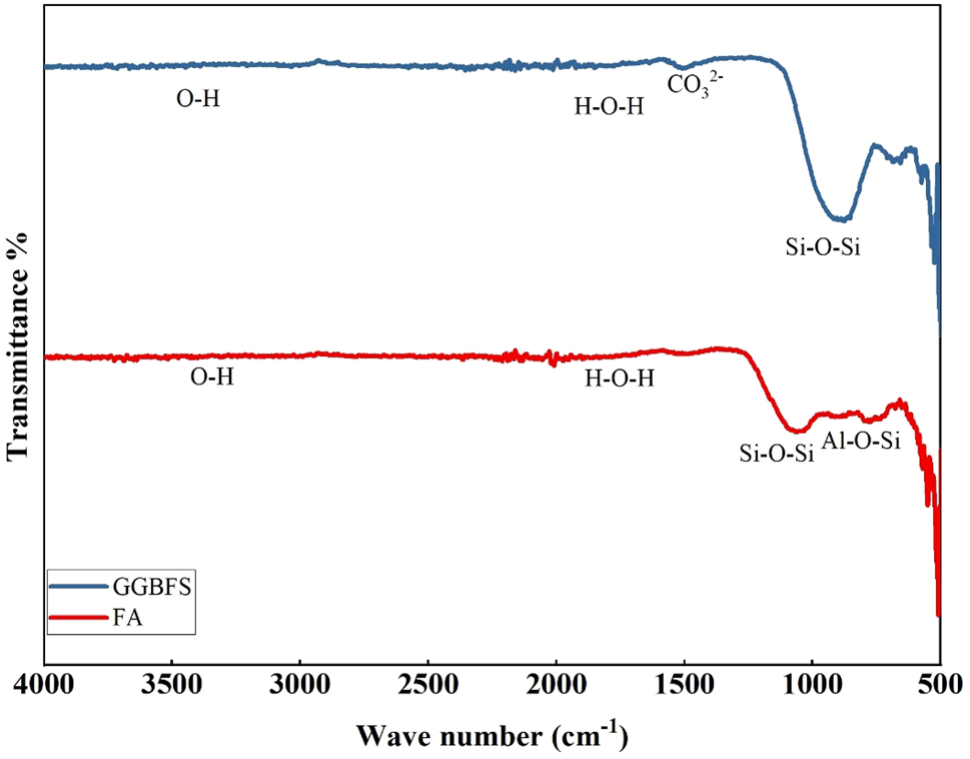
FTIR image of GGBFS and FA.

This section briefly describes the materials employed in the current investigation. The binding materials comprise a combination of GGBFS and FA, with GGBFS and FA being significant parts of the total binder. Astraa Chemicals in Chennai supplied the GGBFS, while the FA was obtained from the Ramagundam Thermal Power Plant in Telangana. The chemical analysis revealed that GGBFS consists of 35.37% lime (CaO), 33.06% silica (SiO_2_), and 16.81% alumina (Al_2_O_3_) as major components, with the rest being minor ingredients. The chemical analysis of FA showed 48.81% silica, 3.80% lime, and 31.4% alumina. The Blaine’s fineness of GGBFS and FA was measured at 390 m^2^/kg and 327 m^2^/kg, respectively. The specific gravity of GGBFS is 2.85 and that of FA is 2.04. All these material qualities meet the criteria set by ASTM C618 and BS EN 15167-1^36,37^. The FTIR analysis of FA and GGBFS used in the study are depicted in Fig. 1. It shows that the range of wavelengths from 3500 to 1600 cm^−1^ indicates the stretching of the (–OH) bonds and the bending vibrations of (H–O–H) in water molecules that are absorbed on the surface and trapped in the spaces of the polymeric structure. The band between 1600 and 1000 cm^−1^ shows that Si–O–Si bonds are present, characteristic of quartz. At about 1470 cm^−1^, the frequencies of CO_3_^–2^ were found to indicate stretching vibrations of the C–O groups. The 900–500 cm^−1^ band indicates that the Al–O–Si and Si–O–Si bonds stretch symmetrically. This transition from amorphous to semi-crystalline alumino-silicate solids occurs. The band seen at frequencies below 500 cm^−1^ consists of the bending waves of the bonds Si–O–Si and O–Si–O.

**Figure 2:**
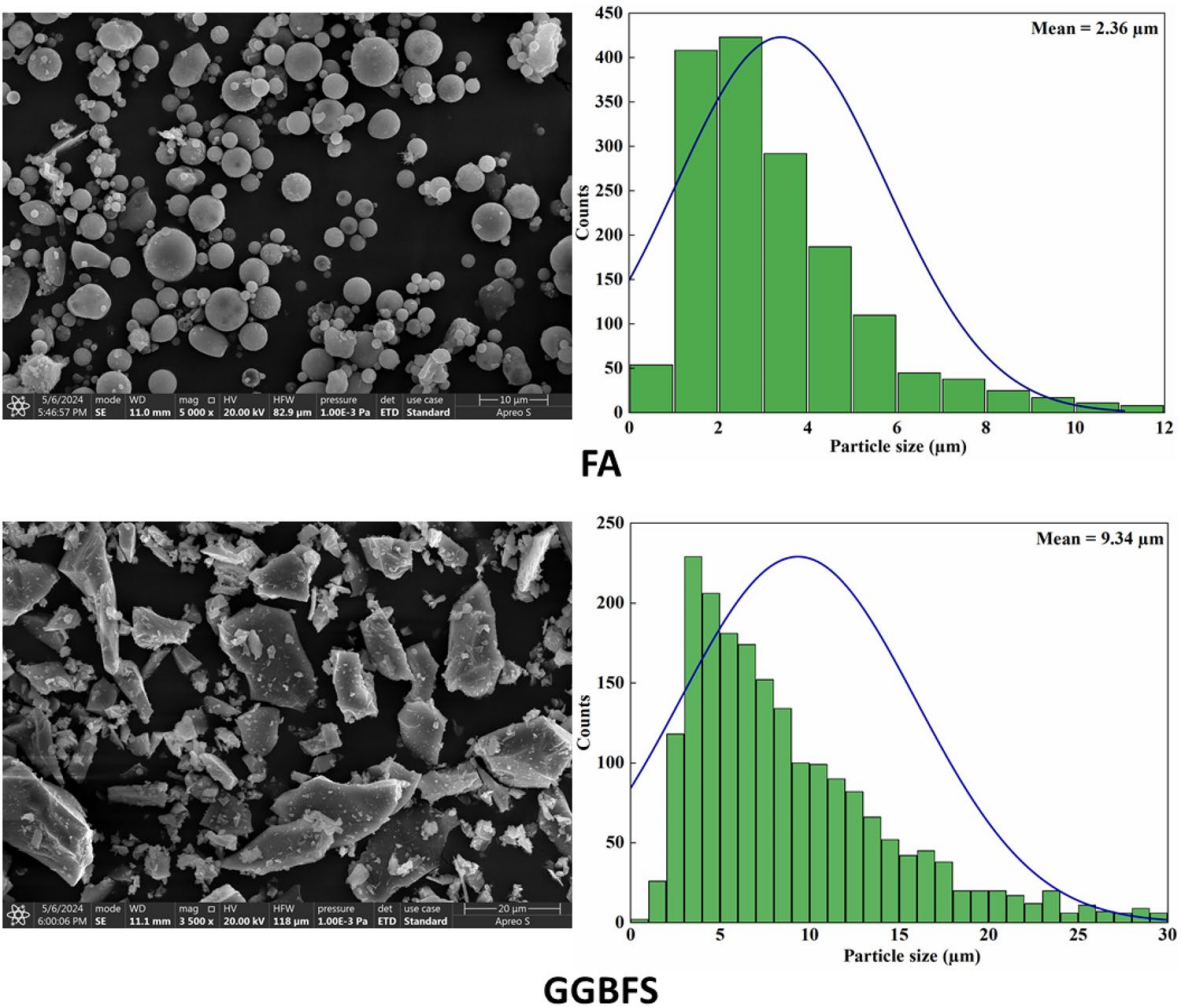
SEM image and average particle size of FA and GGBFS.

The SEM images and average particle size distribution of binders (i.e., GGBFS and FA) used in the study are illustrated in Fig. 2. It depicts that the particles of GGBFS have sharper, denser, and more irregular crystal structures. On the other hand, FA particles primarily display a spherical morphology. The particle size distribution of the binders is shown in Fig. 2. The measurements obtained from ImageJ software show that approximately 90% of particles are in a range of 0.5–10 µm and 0.11–30 µm for FA and GGBFS, respectively. The histogram illustrates that the mean particle size of FA and GGBFS were 2.36 µm and 9.34 µm, respectively.

The locally accessible quarry river sand that satisfied IS 2386^38^ requirements for Zone-II grading and had a specific gravity of 2.56 was employed for the study. The coarse aggregates for the reference mix were crushed granite stones procured from a local quarry. For the other combinations, the CRAs were obtained from the M/s Re Sustainability Limited, Hyderabad recycling plant; the obtained CRAs were sieved to remove particles larger than 20 mm. As per the previous research by Khan et al.^35^, PCRAs were made by subjecting CRAs to a hybrid pre-treatment method (i.e., chemical pre-treatment with a 0.1 M CRCFS-1500 acidic solution followed by a short mechanical pre-treatment process). A crushed angular NA, CRAs and PCRAs with a specific gravity of 2.67, 2.34 and 2.62, respectively, and water absorption rates of 0.26, 4.67, and 0.87, respectively, and a size passing through a 20 mm and held on a 4.75 mm mesh were adopted, meeting the requirements as per IS 2386^38^. The available tap water was used to prepare the AAC mixes. It was ensured that it was free of any impurities as per the requirements of IS 456^39^.

NaOH solid flakes and Na_2_SiO_3_ solution were used as the AAS. The supplier of both compounds was M/s Amrutha Organics in Hyderabad, India. As per IS 14212^40^, the composition of Na_2_SiO_3_ was ascertained. It was found that 0.094 kg of Na_2_O, 0.301 g of SiO_2_, and 0.605 kg of H_2_O constitute 1 kg of Na_2_SiO_3_ solution. Na_2_SiO_3_ was discovered to have a Ms value of 3.20. After dissolving NaOH in H_2_O to develop the AAS, Na_2_SiO_3_ was added to the mixture to get the desired modulus (Ms = 1.25). Based on the literature, the activator solution was adjusted to yield a 4% dosage of Na_2_O (by weight of total binder content) with a set Ms value of 1.25^41,42^. The solution was mixed thoroughly and transferred to a closed container. It was left for one day before being used in the AAC. The w/b ratio of 0.45 was attained by adding more water.

An air-entraining agent would be beneficial when mixing the AAC with different coarse aggregates, as identified from the previous work^43^. Therefore, the present study used an air-entraining agent, i.e., MASTERAIR 721, to prepare the AAC. The local supplier provides the chemical properties of AEA (i.e., the pH, relative density, chloride ion content, and aspect are 6, 1.020, < 0.2%, and pink free-flowing liquid, respectively).

### Sample preparation

The design mix for AAC pavements was designed using the method recommended for standard OPC mix design-based Pavement Quality Concrete. The study involved preparing a mix design with a total cementitious content of 400 kg/m^3^ while keeping a water-to-binder ratio of 0.45 and an acceptable fine aggregate-to-coarse aggregate ratio of 0.35:0.65. According to the previous work^35^, the AAC blends were made using GGBFS and FA binders in a 40:60 ratio. The total amount of water in the AAC mixes included the water that was already in the Na_2_SiO_3_ solution plus the extra water that was added to achieve the desired consistency. The quantum of NA was systematically varied in the combination by incorporating CRAs and PCRAs; the mix design details are presented in Table 1.

**Table 1:** Summary of the combination of CRAs and PCRAs-based AAC specimens (kg/m^3^).

Mix Labels	Binders (400 kg/m^3^)		Sand (kg/m^3^)	Coarse aggregates (1172 kg/m^3^)			Na_2_SiO_3_ (kg/m^3^)	NaOH (kg/m^3^)	Water content (kg/m^3^)
	GGBFS @ 60%	FA @ 40%		NA	CRAs	PCRAs			
AAC	160	240	605	100%	–	–	66	13	99
AACR1	160	240	605	80%	20%	–	66	13	99
AACR2	160	240	605	60%	40%	–	66	13	99
AACR3	160	240	605	40%	60%	–	66	13	99
AACR4	160	240	605	20%	80%	–	66	13	99
AACR5	160	240	605	–	100%	–	66	13	99
AACT1	160	240	605	80%	–	20%	66	13	99
AACT2	160	240	605	60%	–	40%	66	13	99
AACT3	160	240	605	40%	–	60%	66	13	99
AACT4	160	240	605	20%	–	80%	66	13	99
AACT5	160	240	605	–	–	100%	66	13	99

The AAC was prepared using a traditional method and then placed into the requisite moulds in three layers to ensure proper compaction through the needle vibrators. The samples were let to settle under standard laboratory conditions for about 24 h. Then the AAC samples were left to ambient curing at room temperature until testing.

### Test programs

**Figure 3:**
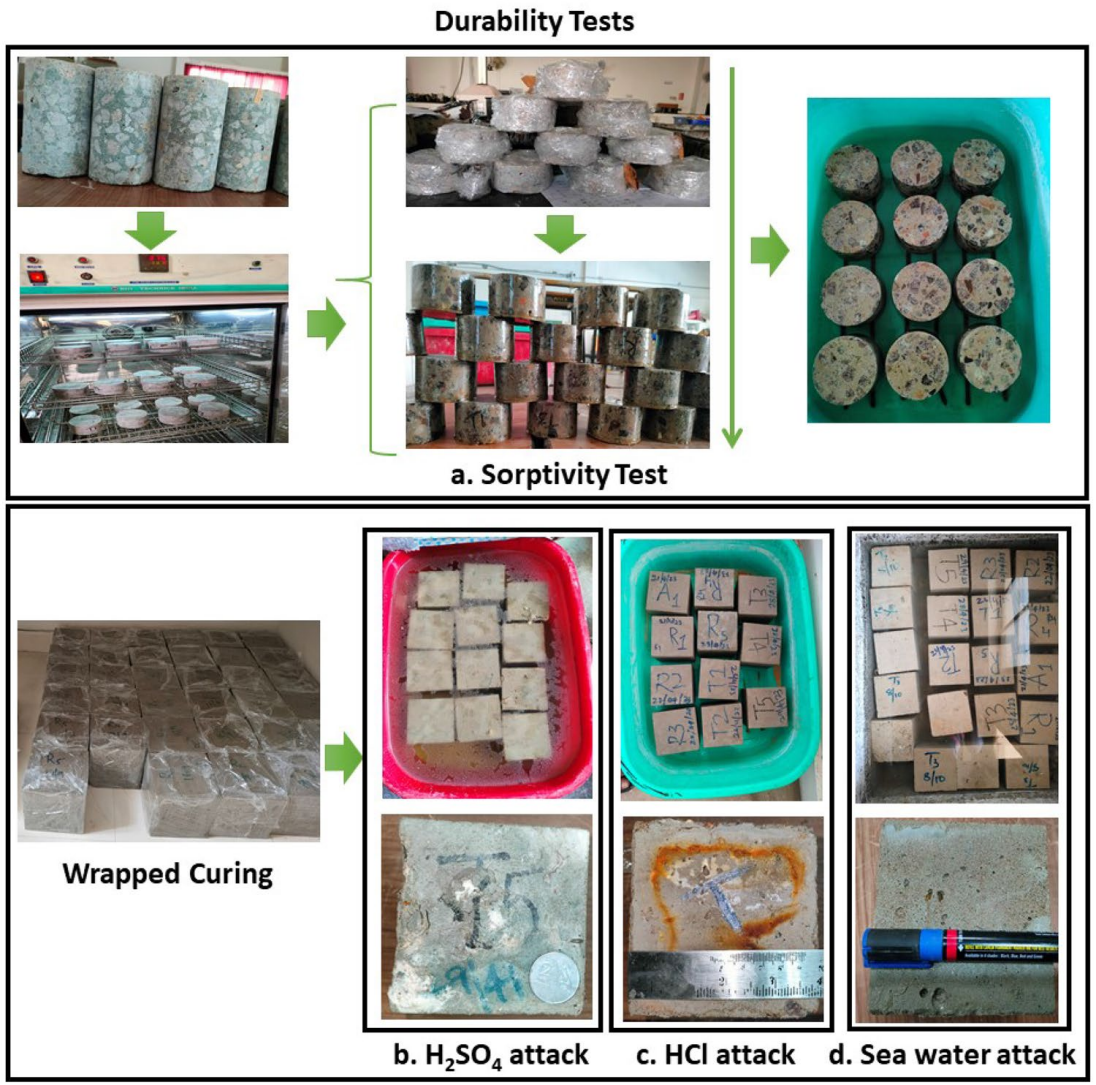
Experimental work adopted for present work.

The AAC specimens were subjected to several examinations to determine their durability and microstructural properties. The compressive strength of the AAC samples was determined using the standard cubes of 100 mm size. Following IS 516^44^, these tests were conducted following ambient curing. Observing the recommendations in ASTM-C1585^45^, this experiment sought to quantify the rate at which a specimen absorbs water (sorptivity), with only one surface of the AAC specimens in contact with water. The AAC specimens were paced in a relative humidity chamber at 50 ± 2 °C for 3 days. After that, they were tightly sealed in polythene bags and kept at 23 ± 2 °C for 15 days. To find the initial mass of the samples, the side surfaces were sealed with epoxy glue, and the samples were weighed. Water was added to the sealed specimens in a plastic container 3 mm above the supports. The AAC samples that were put into the container are displayed in Fig. 3. The AAC sample mass was recorded, and from the expression I = m_t_/(a × d), the sorptivity coefficient (S) is determined as per ASTM-C1585. Where I denote absorption (mm) and t represents time (s). This test follows ASTM-C1898^46^, to assess acid attack on AAC specimens in an acid solution (i.e., HCl and H_2_SO_4_). The AAC specimens were removed after curing 28 days, allowed to dry for 16–18 h, and were weighed. To accomplish this test, 5% of HCl and H_2_SO_4_ were prepared and diluted to a pH of about 1.5–2. AAC specimens were immersed in two acidic solutions for 56 and 90 days (Fig. 2). To maintain its pH, the acidic solution was examined once a week. The specimens were removed after 56 and 90 days (Fig. 3). The specimens underwent cleaning to remove acid-leached unstable particles, final weight measurement, and compression testing. The percentage of loss in strength was obtained.

A similar procedure and timeline were adopted to assess the seawater resistance of AAC specimens with seawater obtained from the Bay of Bengal, India. The pH of seawater is 8.1, the conductivity is 30.6 mS/cm, the total dissolved solids are 15.3 PPM, and the turbidity is 0.49 NTU, respectively. After ambient curing for 28 days, the AAC samples were placed into a container of seawater, which was constantly changed every 15 days to maintain the concentration of the solution. The seawater-exposed samples were tested for compressive strength analysis after 56 and 90 days, respectively.

At 28, 56, and 90 days, for XRD, FESEM and FTIR tests, cube specimens of 100 mm size were fractured using an automatic compressive testing machine and subsequently finely powdered. Selected powder samples before and after various durability tests used for the XRD, FESEM and FTIR tests were kept in an oven for 6–10 h @ 100 °C to stop further hydration. XRD patterns were obtained using a ULIMA IV Rigaku instrument with a scanning range from 10° to 70° at a step size 0.01°. The FTIR-4200 spectra were acquired using a Ge/KBr beam splitter with a precision of 0.01 cm^−1^, covering the 4000 to 500 cm^−1^ range. The spectra were detected using a DLATGS detector manufactured by Jasco. The microstructure of powdered samples was investigated using Oxford Instruments EDX analysis equipment and high-resolution SEM (APEROS, FEI). The surface characteristics of AAC, AACR5 and AACT5 samples were investigated using a stereomicroscope system from Olympus.

## Results and discussions

### Compressive strength

**Figure 4:**
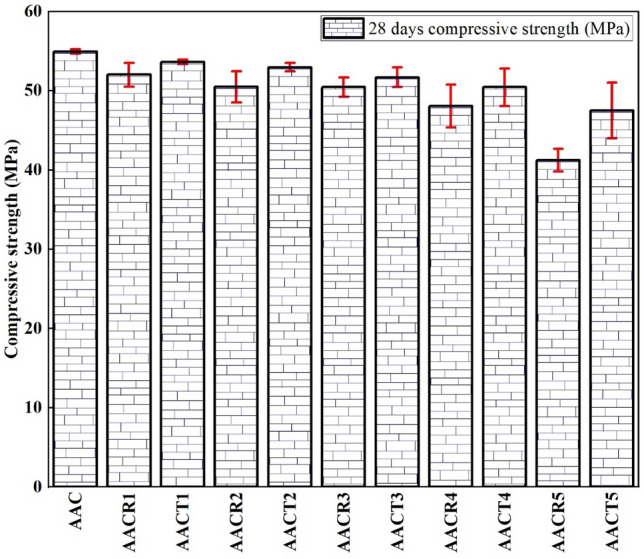
Compressive strength of the mix.

The cube specimens of AAC with NA, CRAs and PCRAs were examined to obtain the compressive strength at 28 days. Figure 4 displays the test findings. According to the findings, the PCRAs efficiently enhance compressive strength. The compressive strength of the AAC with NA was 55 MPa at 28 days. If 20% of NA was replaced with CRA (i.e., AACR1), the compressive strength decreased to 52 MPa at 28 days. However, if this substitute was PCRAs, the compressive strength of AACT1 reached 53 MPa at 28 days. In this sense, using PCRAs almost compensated for the strength reduction as the supplementary material to NA.

In addition, for the 100% replacement ratio, AAC with CRA (i.e., AACR5) exhibited a strength of 41 MPa at 28 days. There was a sharp fall in compressive strength if the NA was replaced with unprocessed CRA. Even when using 100% PCRA instead of CRA (i.e., AACT5), exhibiting a strength of 47 MPa at 28 days still apparently lower than that of the AAC with NA. Hence, using 100% PCRAs should be considered a compromise to the fall in compressive strength. The compressive strength of the AACR5 and AACT5 achieved strengths of 75% and 86%, respectively, compared to that of AAC with NA at 28 days. This is due to the high porosity and water absorption in all the AAC with CRAs samples, which further reacts and loses strength^47,48^. However, due to the pre-treatment process, AAC with PCRAs attained more strength than AAC with CRAs^35^.

### Sorptivity

**Figure 5:**
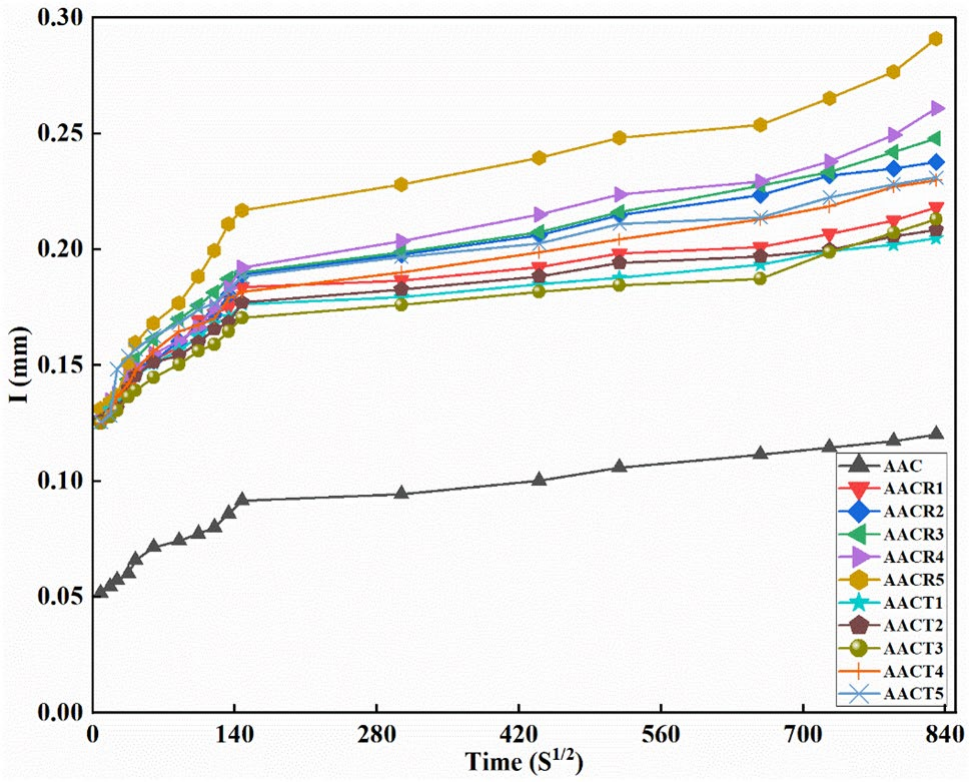
Sorptivity analysis of the mix.

The ability of a concrete surface to absorb water is affected by several elements, including the types of concrete mixes used, chemical additives, additional cementitious materials, entrained air, curing type and duration, rate of hydration, concrete age, the presence of water, and others. The sorptivity graphs obtained for the AAC with varying NA, CRA and PCRA amounts are shown in Fig. 5. Similar to the AAC made of NA, the AAC blends’ absorption rate with PCRAs also decreased over time, and there was no meaningful increase in the absorption rate after 240 min for most of the samples. This is due to the improved matrix or gels in AAC compared to CC. It has also been noted that the amount of absorption increases with the increased CRA amount. The behaviour of PCRAs was similar to that of the AAC made of NA.

As seen in Fig. 5, the sorptivity coefficient increased gradually with the percentage of unprocessed CRA ranging from 0 to 100% in AAC. The absorption of the AAC with NA was 0.19 mm at the final stage. If 100% of NA was replaced with CRA (i.e., AACR5), the absorption increased to 0.29 at the same period. However, if this substitute was PCRAs, the compressive strength of AACT5 reached 0.23 at its final stage. In this sense, using PCRAs has almost overcome the sorptivity as the alternative material to NA. Therefore, incorporating CRAs as alternative aggregates in AAC may introduce incomplete reactions due to high water absorption and porous nature associated with CRAs, which can increase sorptivity.

Moreover, the absorption rate remained constant with the increase in the soaking period, mainly due to the minimal heat of hydration in AAC compared to CC. Similar kinds of absorption observations have been reported for the AAC blends^8^.

### Hydrochloric acid resistance

**Figure 6:**
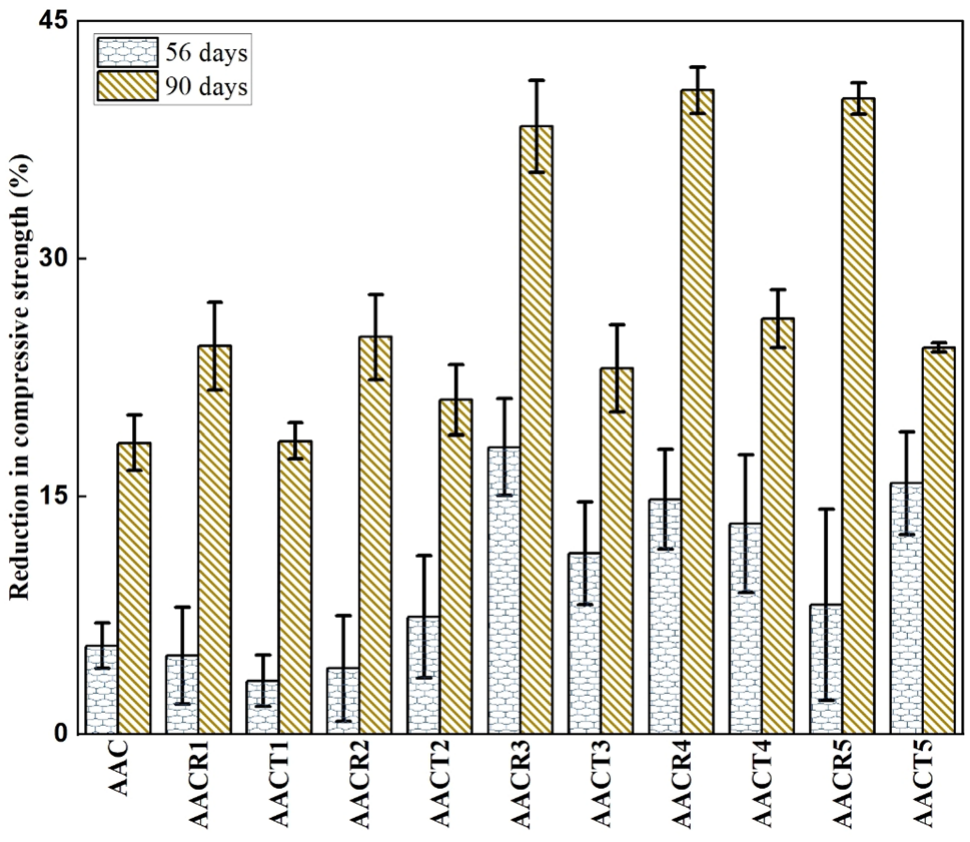
Residual compressive strength of samples after exposed to HCl.

Figure 6 illustrates the apparent deteriorating behaviour of AAC with NA, CRAs, and PCRAs when submerged in HCl solution. The gels degradation occurs due to a reaction between the H^+^ ions in the acid and the gels (CASH and NASH) in the AAB. CaCl_2_, a highly soluble salt, forms when the matrix’s HCl and calcium combine. CaCl_2_ increases the porosity of the AAC sample by leaking out of the pore channels. Following 56 and 90 days, respectively, of acid soaking, the compressive strength of the AAC samples likewise dramatically drops (Fig. 6). Compressive strength is still higher in the AAC with 100% PCRA samples than in the AAC with 100% CRA samples, but both CRAs and PCRA-based AAC have lower strength than the AAC with NA samples. All CRA samples do, however, have comparatively high strength-loss ratios. The CRA samples in the AAC with attached mortar and old ITZs phase progressively react and lose strength after prolonged soaking^18^. The pre-treatment procedure that improves the CRAs qualities is why the AAC containing PCRAs has a comparatively high strength following acid soaking. It must be acknowledged that the incorporation of PCRAs into the AAC benefits its HCl acid resistance.

After undergoing sulfuric acid corrosion, the compressive strength of the AAC with CRAs reduced from 49 to 38 MPa, the AAC with PCRAs from 52 to 40 MPa, and the AAC with NA from 55 to 52 MPa over 56 days. The strength further decreased as the soaking period was extended from 56 to 90 days. AAC with CRAs dropped from 39 to 25 MPa, the AAC with PCRAs from 44 to 36 MPa, and the AAC with NA from 52 to 45 MPa over 90 days.

### Sulfuric acid resistance

**Figure 7:**
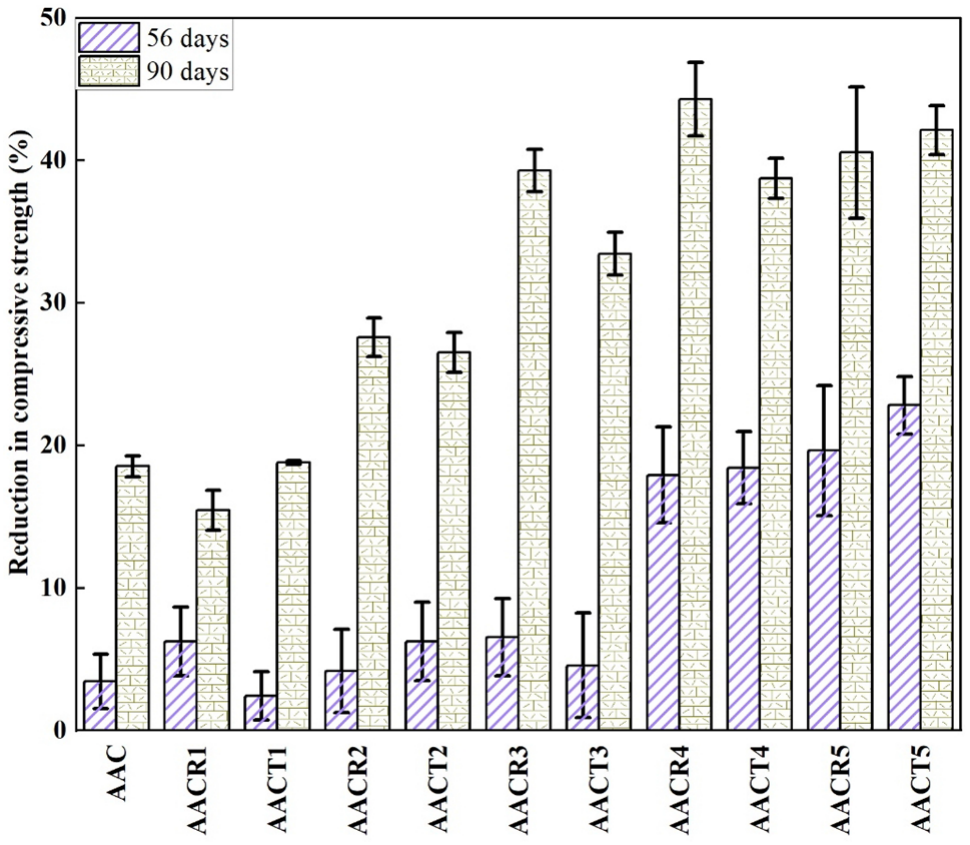
Residual compressive strength of samples after exposed to H_2_SO_4_.

As illustrated in Fig. 7, H_2_SO_4_ has a more noticeable corrosion effect on all AAC samples when compared to other acids. The AAC expands and cracks due to the growth, precipitation, and production of gypsum crystals under the influence of H_2_SO_4_. The primary source of the decrease in strength is the dehydration of ettringite, which leaves behind a white residue on the sample’s surface consisting of gypsum and ettringite. After the AAC samples are soaked in the H_2_SO_4_ solution, the calcium, alumina, sodium, magnesium, and potassium ions transfer into the H_2_SO_4_ solution. In contrast, the SO_4_^2-^, H_3_O^+^, and H^+^ ions penetrate the sample, leading to a decline in strength.

The compressive strength of the AAC with CRAs dropped from 49 to 33 MPa after 56 days of sulfuric acid corrosion. It dropped from 52 to 37 MPa for the AAC with PCRAs and from 55 to 53 MPa for the AAC with NA. The strength decreased as the immersion period increased from 56 to 90 days^49^. AAC with CRAs dropped from 44 to 25 MPa, the AAC with PCRAs from 44 to 28 MPa, and the AAC with NA from 55 to 45 MPa for 90 days.

### Seawater resistance

**Figure 8:**
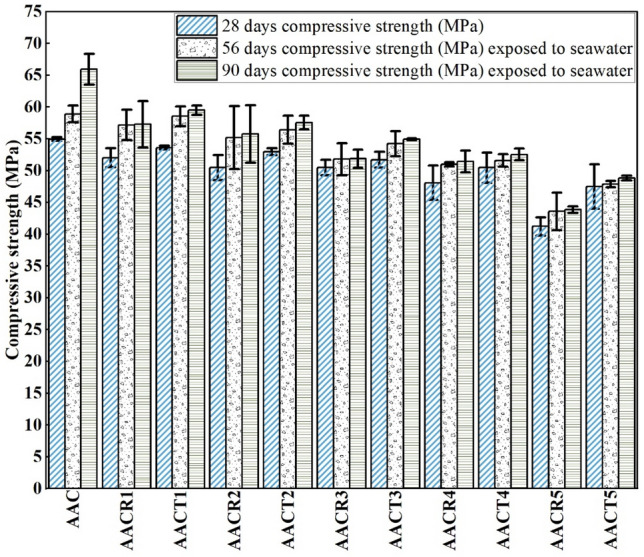
Compressive strength of samples before and after exposed to seawater.

In contrast to earlier studies^50–52^, the compressive strength of AAC with NA, CRAs, and PCRAs increased after being submerged in seawater for 56 and 90 days. This enhancement is shown in Fig. 8. The improvement of the microstructure was caused by the ongoing activation process (pozzolanic reactions, reaction–diffusion) and the formation of crystalline hydration products of calcium silicates. In addition to the continuing infiltration of hydration products into the pores, which leads to greater structural density, previous research has also linked the rise in strength to the expansion of internal elements such as calcium, improving the composite samples’ overall integrity. The increase in strength persists until the pressure caused by the expanding ettringite surpasses the available space, leading to the development of internal micro-cracks and a subsequent decrease in strength.

The relatively lesser average gain in compressive strength of 57.1 MPa, 55.2 MPa, 51.7 MPa, 50.7 MPa and 43.6 MPa for variation of 0–100%, respectively, was obtained for AAC with CRAs due to higher porosity and absorption compared to 58.5 MPa, 56.4 MPa, 54.2 MPa, 51.8 MPa and 47.8 MPa gain for AAC with PCRAs when exposed to seawater for 56 days, respectively. Whereas the lower average gain in compressive strength 57.3 MPa, 55.7 MPa, 52 MPa, 50.8 MPa and 43.8 MPa for variation of 0–100%, respectively, was obtained for AAC with CRAs due to adhered mortar than 60 MPa, 57.5 MPa, 54.9 MPa, 52.5 MPa and 48.8 MPa gain for AAC with PCRAs when exposed to seawater for 90 days, respectively^15,19,53^. The higher average strength gain was recorded for AAC with 59 MPa and 66 MPa NA specimens for 56 and 90 days, respectively. The adhered mortars on CRAs increase the porosity and water absorption that introduce new ITZs into a matrix and weaken it. In contrast, the pre-treatment process of CRAs densifies the matrix and restricts different ITZ zones. This phenomenon results in lower strength gain for AAC with CRAs than for AAC with PCRAs.

## XRD analysis

**Figure 9:**
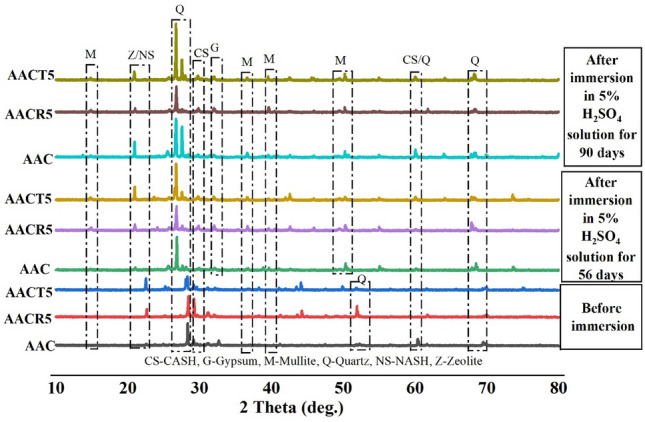
XRD analysis of samples exposed to H_2_SO_4_.

**Figure 10:**
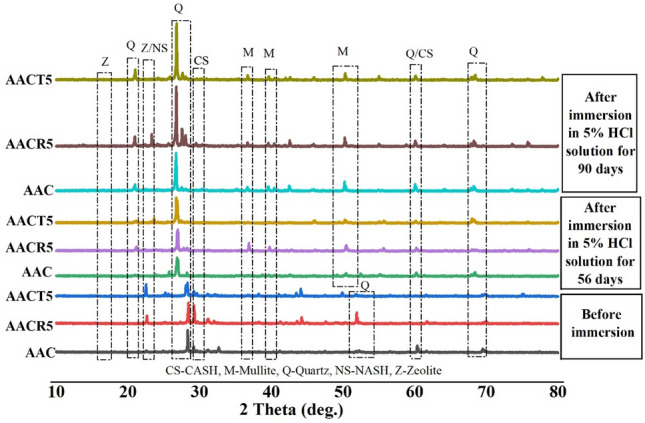
XRD analysis of samples exposed to HCl.

**Figure 11:**
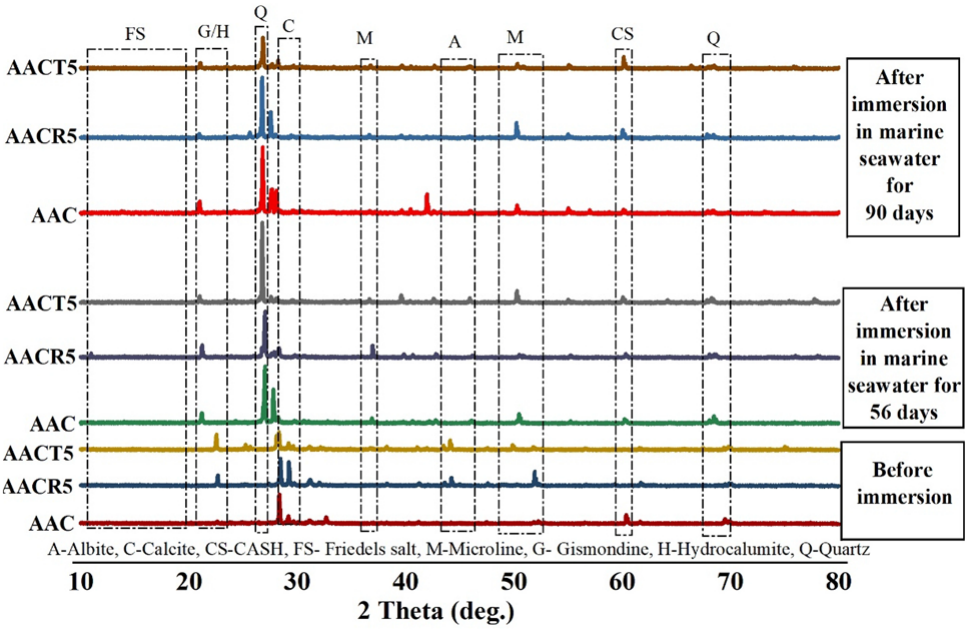
XRD analysis of samples exposed to seawater.

Figures 9, 10 and 11 depicts the XRD pattern with peak phases of AAC samples with CRAs, PCRAs, and NA. The pattern is shown before and after exposure to marine seawater, HCl, and H_2_SO_4_ solutions. X-ray diffraction (XRD) examination was conducted on AAC samples made from 100% CRAs and PCRAs, as well as conventional samples made from AAC with 100% NA, as shown in Table 1. Singh et al.^26^ conducted an X-ray diffraction (XRD) examination on the unexposed specimens used in the present study. Crystalline quartz, mullite, and calcium-based compounds, specifically calcium aluminium silicate hydrate (CASH), were the primary phases observed in the selected AAC samples (i.e., AAC, AACR5 and AACT5) before being exposed to seawater and acid solutions^54^. Typically, the quartz and mullite phases are identified by examining the unreacted or unhydrated FA particles in the hardened sample. On the other hand, the hydration reaction produces CASH as the resulting product. The CASH phase was present with the major reaction product CSH, known as gismondine. The detection of the zeolite phase at around 16–17° of 2θ confirms the existence of NASH. However, the intensity of this phase was significantly weak. On the other hand, the extra Si and Ca ions from the binders (GGBFS and FA) led to more primary reaction products by reducing the amount of unhydrated compounds that were present. The XRD findings from this investigation corroborate the 28-day compressive strength of the AAC samples shown in Fig. 4, as do the results of other studies that have been published^55–57^.

As demonstrated in Fig. 9, all of the samples showed the presence of crystalline phases of unhydrated substances (quartz and mullite) and a new crystalline gypsum phase (CaSO_4_·2H_2_O) after being exposed to a 5% H_2_SO_4_ solution. The crystalline phases of gypsum, quartz, and mullite replaced the hydrated phases (CASH and NAS-H) present in the samples before acid assault after exposure to 5% H_2_SO_4_ acid. The observed shift in reaction products after immersion in the H_2_SO_4_ solution suggests that, although the HSO^4-^ ions promoted the formation of crystalline gypsum by reacting with the released Ca^2+^ ions from the precursor materials, the H^+^ ions from the solution weakened the structure of the hydrated phases. This suggests that the Si, Ca, or Na ions present in binders are predominantly utilized in the early stages of the reaction to create a durable structure of aluminosilicate gels. Consequently, the AAC sample’s matrix contained a reduced quantity of Ca ions, which hindered the formation of expanding gypsum crystals upon exposure to an H_2_SO_4_ solution^58^. The AAC with NA mix exhibited the most minimal reduction in strength upon exposure to an H_2_SO_4_ solution. Nevertheless, the magnitudes of these peaks were notably greater in AACR5 in comparison to AACT5. Furthermore, the presence of CRAS in AACR5, which is linked to the amount of mortar adhering to the surface, facilitated the penetration of the acid solution into the material’s structure as a result of its high porosity and water absorption. Consequently, this led to a notable degradation. This corroborates the decline in the structural integrity of the examined mortars following exposure to acid, as discussed in the preceding sections.

Figure 10 illustrates that once the samples are submerged in a 5% HCl solution, the phases seen in the samples before exposure mostly stay the same. Still, certain minor adjustments about the position and size of the peaks are discernible. More specifically, when exposed to HCl, the mullite and crystalline quartz phases changed in composition. In addition, these peaks’ magnitudes are noticeably higher than seen in the samples before exposure. Exposure to a highly acidic environment increases the amount of wholly or partially dissolved binder particles in the matrix, which explains these phenomena. In addition, the peaks of CASH (which coexisted with CSH) and NASH exhibited a change in intensity upon exposure to HCl acid^58^. Prior to the exposure, these peaks were characterized by a broad hump. This suggests that the acid attack gradually breaks down the interconnected polymeric structures, making non-interconnected crystalline phases more accessible in the matrix when it is immersed in an acid solution for an prolonged amount of time. Nevertheless, following exposure to HCl, the magnitudes of the peaks detected in AACT5 were diminished compared to those observed in AACR5. It seems that the pre-treatment process of CRAs, specifically PCRAs, stops the HCl acid attack from breaking down the gel network. The mixture with PCRAs demonstrated better resistance to HCl attack compared to those with CRAs. This is due to the same factors that were mentioned earlier for H_2_SO_4_ attack.

Figure 11 highlights the XRD diffractograms of selected samples (AAC, AACR5, and AACT5) before and after being exposed to marine seawater for 56 and 90 days. The AAC mix exhibits strong peaks of quartz and gismondine as crystalline phases in all blended mixes. The results depicted in Figs. 9 and 10, indicate that the quartz peak remains detectable even when CRAs and PCRAs are included, both before and following exposure to the marine environment. This implies that quartz does not take up any physical space within the polymerization system. The polymerization method in AAB involves a substantial quantity of CSH that is tightly attached to the calcite phase. Peaks in the 29°–30° 2θ range serve as indicators of the phase of interest. It should be noted that the binders, namely GGBFS and FA, expedite the AAC reaction and enhance the rate of hydration. The concentration of C–S–H, calcite, hydrotalcite, gehlenite, and anorthite decreased from 28 to 90 days, whereas the concentration of gismondite and albite increased over this period. In addition, it was noticed that Friedels’ salt was present in small proportions in the samples after 56 and 90 days^15,59^. This, in turn, results in the strengthening of the material, especially in AAC and AACT5, as illustrated in Fig. 8.

## SEM analysis

**Figure 12:**
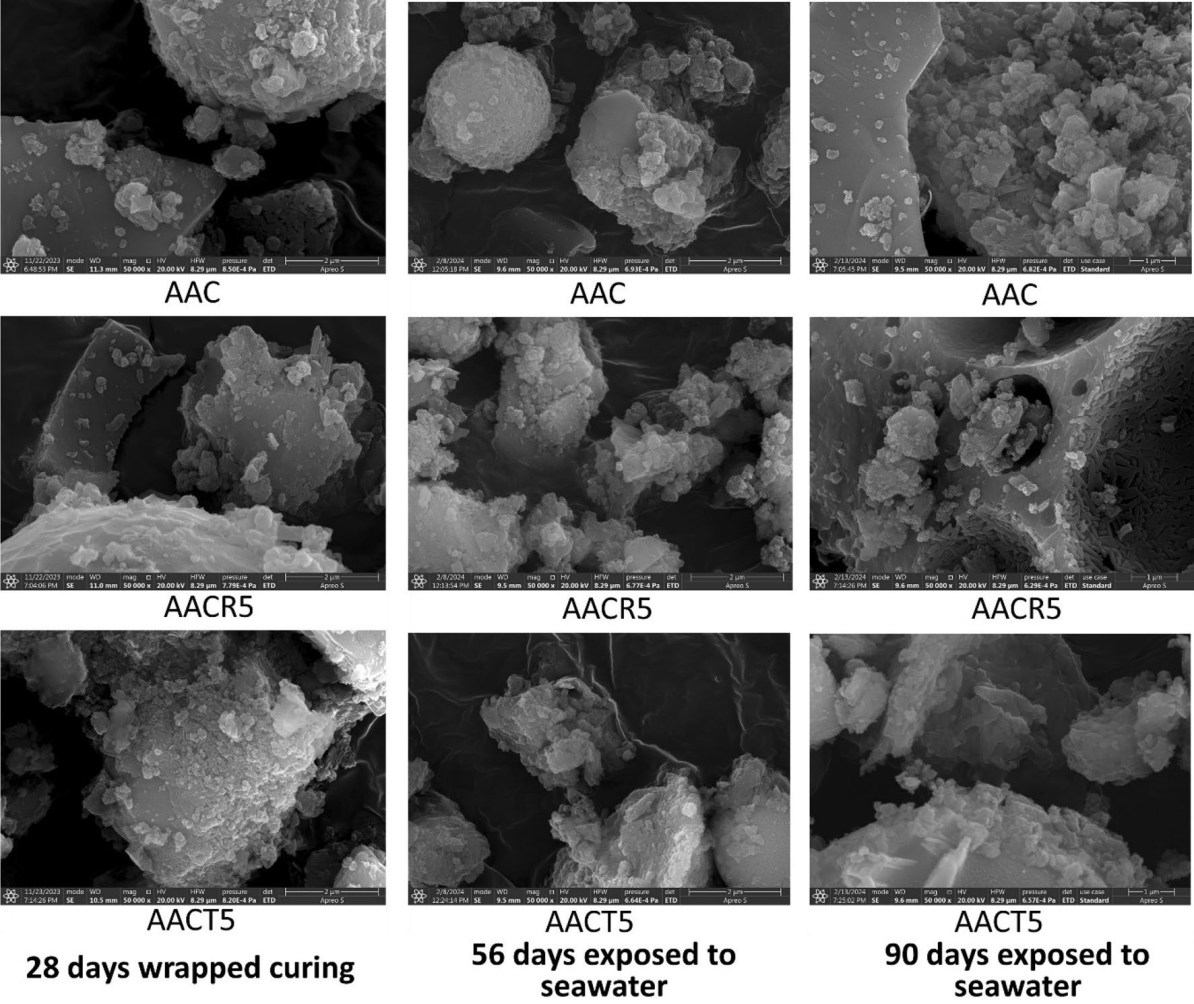
SEM images of samples exposed to seawater.

**Figure 13:**
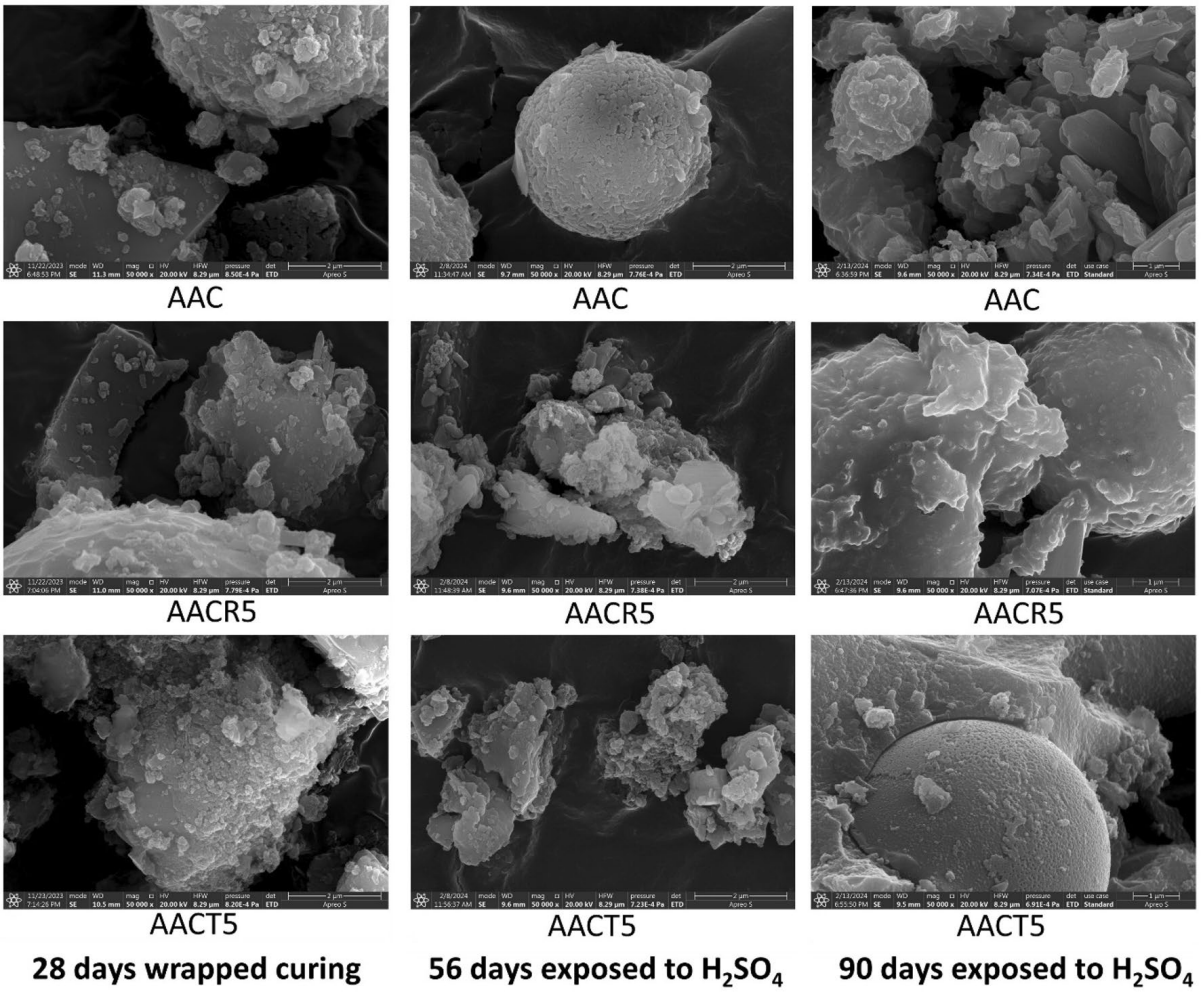
SEM images of samples exposed to H_2_SO_4_.

**Figure 14:**
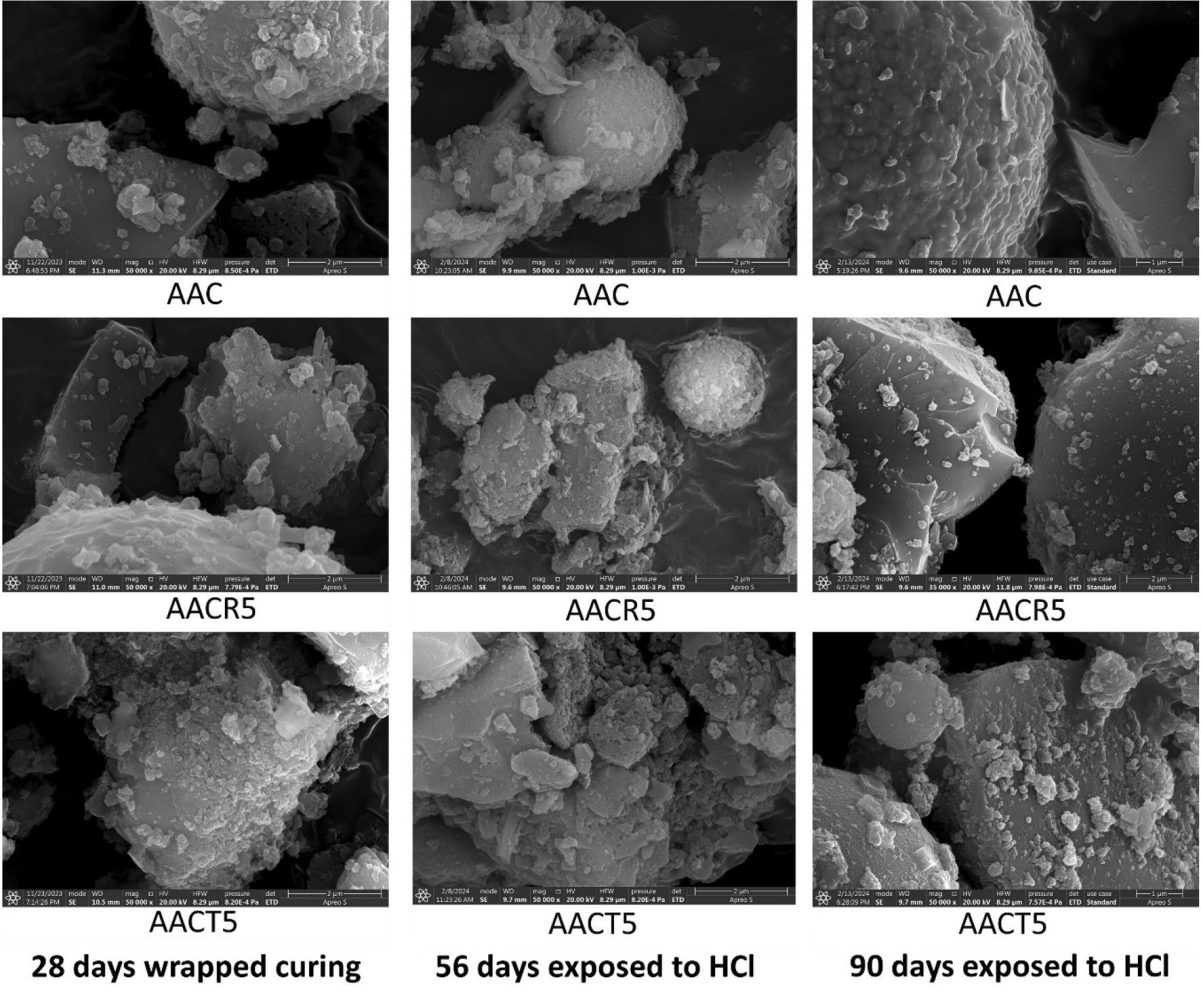
SEM images of samples exposed to HCl.

To better understand the influence of seawater, HCl, and H_2_SO_4_ solutions on the surface morphology features of the selected samples (i.e., AAC, AACR5 and AACT5) images are obtained before and after exposure for 28, 56 and 90 days, respectively, which are shown in Figs. 12, 13 and 14. The SEM images depict a well-dispersed paste with fewer embedded unreacted slag grains (which have an irregular shape), suggesting a denser and more uniform microstructure. The majority of the slag particles have undergone dissolution due to the alkali activators, resulting in the formation of a NASH and CASH gel^19,60–62^. The microstructure of the AACT5 specimens exhibited a smaller number of cavities and microcracks compared to the AACR5 specimens, resulting in decreased porosity and reduced permeability to hazardous ions.

Furthermore, AAB exhibits a greater degree of polymerization, which is attributed to the larger concentration of alumino-silicate gel. Upon careful examination, it was observed that the 28-day-wrapped cured samples shown in Fig. 12 represented a lower degree of homogeneity than their analogous samples. This trend aligns with the significant increase in compressive strength of AAC, which reached a value of 65.9 MPa following exposure to seawater for 90 days. Similar findings were reported by the previous researchers^15,53^.

As shown in Figs. 13 and 14, the SEM pictures indicate that the microstructure of the samples exposed to acid exhibited much more heterogeneity than the microstructure of the samples not exposed to acid. This is because an acidic substance caused the aluminosilicate network to break down, which in turn caused the bonds in the AAB to break down^62–64^. The microstructural damage varied significantly between the samples exposed to H_2_SO_4_ and those that were exposed to HCl. Figure 13 demonstrates that after exposure to H_2_SO_4_, the AAC appears more homogeneous and has a compact structure compared to the AACR5 and AACT5.

In Fig. 13, a group of rod-shaped gypsum crystals can be rarely seen in all three samples after exposure to H_2_SO_4_. However, gypsum crystals are less common in the AAC sample compared to the other two. However, the small amount of sulfur indicates that gypsum crystals have formed to some degree in all of the samples. In contrast, the AACR5 microstructure shows a more significant breakdown than AACT5 due to high water absorption, which leads to substantial strength loss after exposure to H_2_SO_4_. Gypsum was anticipated in SEM images after the sulfuric acid attack^65^.

The HCl attack distorted the solid aluminosilicate network, which led to the formation of a diamond-shaped crystalline residue that could not be dissolved. These solid precipitations were minimal in the AAC samples compared to the AACR5 and AACT5 samples. In addition, the morphology was found to stay the same in AAC. Furthermore, AACR5 microstructures were less dense and more porous due to broken gel clusters and loosely linked particles that had not reacted as much as AACT5. As shown in Fig. 14, the HCl-exposed samples had similar elemental traces and levels at the binder phase as the H_2_SO_4_ exposed samples, except that the H_2_SO_4_ exposed samples had a noticeable trace of sulfur. According to Mohseni et al.^66^, the strength loss of samples exposed to HCl is primarily due to the highly soluble and harmful CaCl_2_ salt formed when the samples were submerged in HCl solution.

## Stereomicroscope analysis

**Figure 15:**
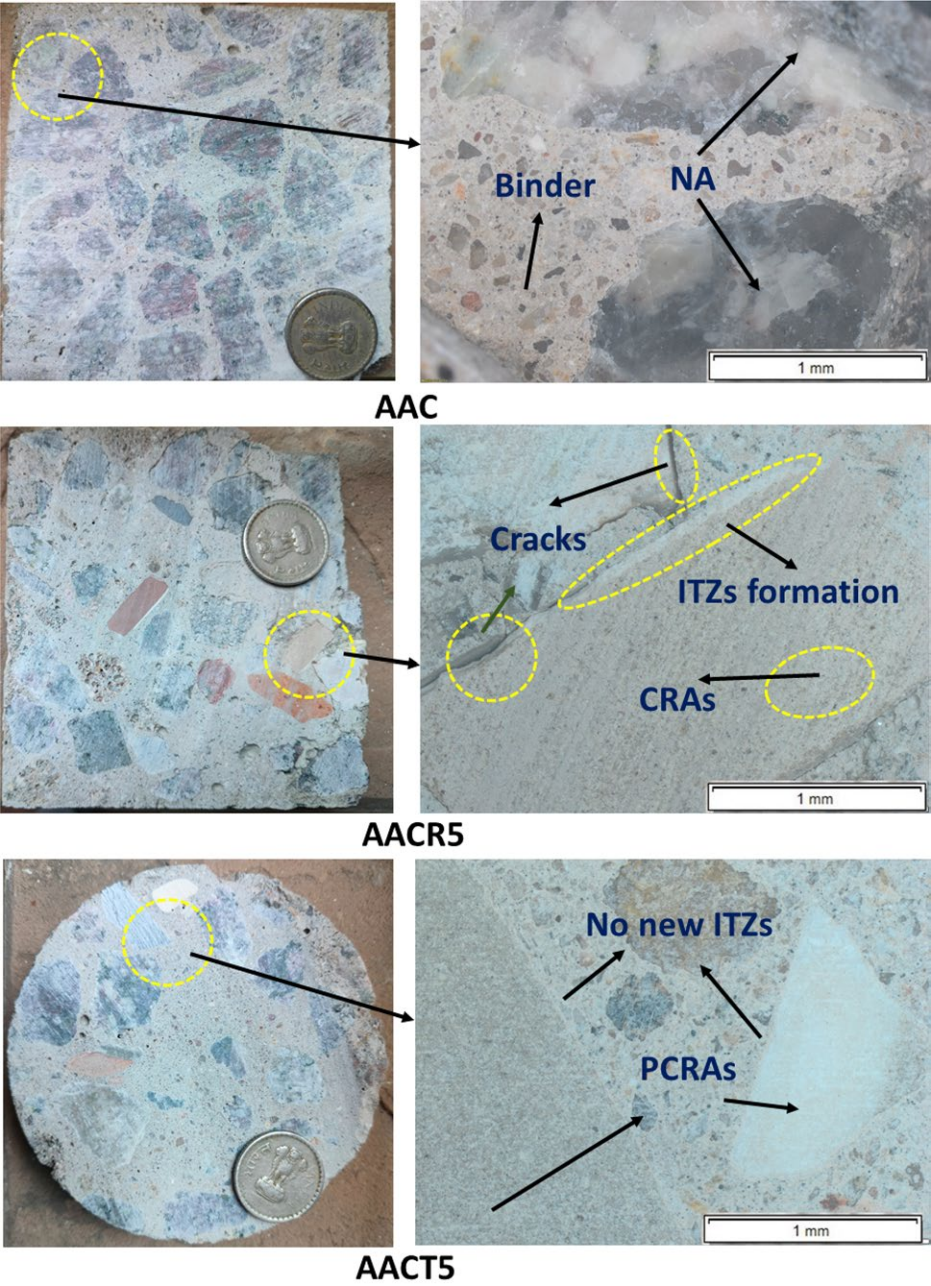
Stereomicroscopic images of AAC, AACR5 and AACT5 samples.

The 100 mm cubes of AAC, AACR5 and AACT5, after 28 days of curing, were sliced and examined under a stereomicroscope. Figure 15 shows the stereomicroscopic images of AAC, AACR5 and AACT5. Visual inspection of AACR5 in Fig. 15 reveals new ITZs, cracks and tiny pores, indicating the presence of old mortar attached to recycling plant-based CRAs. These properties make the recycling plant-based CRAs less suitable for direct use in AAC as they make the mix less potential, reducing its strength and durability, as shown in Figs. 4, 5, 6 and 7. Conversely, AACT5 does not exhibit the formation of additional ITZs. Still, it shows the influence of pretreated CRAs in the same mix, leading to lower performance than AAC with 100% NA but better than recycling plant-based CRAs, as illustrated in Figs. 4, 5, 6 and 7.

## Conclusions

The effects of various coarse aggregates (i.e., NA, CRAs, and PCRAs) with AAC were evaluated for compressive strengths and durability properties like sorptivity, acid and seawater attack tests. The microstructural analysis (i.e., FTIR, FESEM, XRD and stereomicroscopy) was conducted on the samples before and after various durability tests. The following conclusions were drawn based on the results of the specimens provided herein in this study:

- The increase in the replacement percentage of plant-based CRAs in the AAC considerably increased sorptivity due to adhered mortar and higher porosity. Meanwhile, the sorptivity of the AAC with PCRAs was similar to that of the reference mix (i.e., the AAC with NA) and lesser than unprocessed CRAs. This happened due to the pre-treatment of CRAs, which removed the adhered mortar, making the AAC with PCRAs more durable.
- Exposure to H_2_SO_4_ solution for 56 days induced strength losses in AAC-CRAs (6.26–19.65%), and the corresponding overall losses in compressive strength for 90 days (15.45–44.3%) were significantly higher than those of AAC-PCRAs (2.44–18.44%) for 56 days and (18.8–38.75%) for 90 days, respectively.
- Exposure to HCl solution for 56 days induced strength losses in AAC-CRAs (4.97–18.12%), and the corresponding overall losses in compressive strength for 90 days (24.47–40.63%) were significantly higher than those of AAC-PCRAs (3.38–15.85%) for 56 days and (18.5–26.6%) for 90 days, respectively.
- Results of the marine seawater attack for 56 and 90 days showed no deterioration and visible cracks on the overall size and shape of AAC samples. Mainly, GGBFS contributed significantly to boosting the kinetics of the AAB with various coarse aggregates and forming more lower-frequency aluminosilicate-based phases, leading to strength enhancement.
- XRD and SEM analysis support the above findings and indicate that the combined use of GGBFS and FA showed excellent compressive strength and durability properties of AAC by coexisting with CASH and NASH gel, compensating for the adverse effects of using 100% plant-based CRAs.
- The stereomicroscopic images show that AACR5 has a porous microstructure with more prominent voids due to adhered mortar, whereas AACT5 have a better microstructure than AACR5.

In summary, using PCRAs in AAC production promises better durability and performance. Therefore, optimized incorporation of these binders and PCRAs enhances AAC’s durability compared to plant-based CRAs.

### Recommendations

This study potentially motivates upcoming researchers to create multiscale models for assessing and forecasting methods to correlate with the properties of AACs using varying proportions of plant-based CRAs and PCRAs in various environments. This study also motivates future practitioners to find an alternative to alkali-activated solutions like sodium hydroxide and sodium silicates, which can harm health; future studies could focus on making better activators that would not harm the environment. In addition, the amorphous-to-crystalline transitional phases make AAC matrices more structurally stable. Hence, more studies are needed to determine how they react to environmental damage. Finally, it is essential to choose the economic optimization parameters for AAC with CRAs and PCRAs, like life cycle assessments, to use them on a large scale in real life.

## Data Availability

The datasets used and/or analyzed during the current investigation are available from the corresponding author upon reasonable request.
